# Inverted U-shaped relationship between serum vitamin B12 and α-Klotho levels in US adults: a cross-sectional study

**DOI:** 10.3389/fnut.2024.1473196

**Published:** 2024-10-23

**Authors:** Yu-shan Li, Xing-ji Gong, Wen-jie Du, Yang Li, Dong-yong He, Jian Yao, Cui Bai

**Affiliations:** ^1^Department of Emergency Internal Medicine, The Affiliated Hospital of Qingdao University, Qingdao, Shandong, China; ^2^Department of Pediatrics, The Affiliated Hospital of Qingdao University, Qingdao, Shandong, China

**Keywords:** vitamin B12, cobalamin, α-Klotho, homocysteine, oxidative stress, DNA methylation

## Abstract

**Background:**

Serum vitamin B12 and α-Klotho are important markers associated with aging. Limited studies have been conducted on the relationship between vitamin B12 and α-Klotho.

**Objectives:**

This study investigated the relationship between circulating α-Klotho and vitamin B12.

**Methods:**

A total of 4,502 American adults with circulating vitamin B12 levels and α-Klotho levels from the National Health and Nutrition Examination Survey (2011–2014) were included. A weighted multiple linear regression model was used to evaluate the correlation between vitamin B12 and α-Klotho levels. To clarify potential non-linearities, smoothed curve fitting and threshold effects analysis were employed.

**Results:**

A statistically significant non-linear relationship was found between vitamin B12 levels and circulating α-Klotho levels after adjusting for potential confounders. We observed an inverted U-shaped relationship between serum vitamin B12 levels and circulating α-Klotho levels. Notably, serum vitamin B12 levels below the threshold (1,020 pg/mL) exhibited a positive correlation with circulating α-Klotho levels (*β* = 0.14, 95% confidence interval (CI): 0.09–0.18, *p* < 0.0001). Conversely, serum vitamin B12 levels above the threshold (1,020 pg/mL) exhibited a negative correlation with circulating α-Klotho levels (*β* = −0.12,95% CI: −0.17−−0.06, *p* < 0.0001). Sensitivity analyses were performed and consistent results were obtained.

**Conclusion:**

This study demonstrated an inverted U-shaped relationship between circulating vitamin B12 and α-Klotho in American adults. The optimal concentration of serum vitamin B12 in American adults was found.

## 1 Introduction

Aging is a complex biological process accompanied by a gradual decline in several physiological and metabolic functions, leading to the development of a variety of age-related diseases, such as hypertension, diabetes mellitus, and cognitive disorders (1–3). With the trend of global aging increasing, it is becoming important to understand the mechanisms of aging and the factors that influence it (3). Studies have shown that aging is closely related to the dysfunction of multiple humoral factors, cells, and tissues, which provides research directions to promote healthy aging (4, 5).

A-Klotho, a multiactive humoral factor, regulates various responses *in vivo*, including oxidative stress, inflammation, and aging (3, 5–8). In humans, age-related diseases lead to a decline in Klotho levels (2–4, 6). Circulating α-Klotho levels can be used as an indicator for the risk of mortality (1, 9, 10). Growth retardation, vascular aging, renal disease, heart disease, lung disease, multiorgan fibrosis, bone loss, cognitive disorder, and life shortening are exhibited by klotho-deficient mice (1, 9). However, overexpression of this gene yields the opposite effect by extending survival time (4). Thus, the level of α-Klotho may be closely related to the aging state of the organism.

Meanwhile, changes in vitamin B12 (B12), an important micronutrient, have been strongly associated with the risk of aging-related diseases (11, 12). It plays an important role in epigenetic regulatory processes such as DNA methylation, synthesis, and repair (9, 13). Furthermore, potent anti-oxidative stress effects are exhibited by B12 (2, 14). B12 deficiency leads to elevated homocysteine and triggers oxidative stress, which accelerates aging and increases the risk of death (15, 16). Age-related diseases, often linked to oxidative stress, are believed to develop due to chronic micronutrient deficiencies (3, 6). Unfortunately, the global prevalence of B12 deficiency, particularly among the elderly, is attributed to insufficient intake due to economic, cultural factors, and malabsorption, which significantly impacts biological aging (7, 17). Beneficial outcomes have been associated with B12 supplementation in inflammatory diseases (11). Surprisingly, an association between B12 overload and increased all-cause mortality has also been established (5). Therefore, understanding the relationship between B12 levels and aging will provide potential strategies for improving the health of older adults.

While studies have indicated a correlation between α-Klotho and B12, this relationship has important clinical implications for understanding the role of B12 in anti-aging (18). However, the specific relationship between α-Klotho and B12 is still not fully explored at this time. The potential role of B12 in the synthesis and function of α-Klotho warrants further investigation. The aim of this study was to assess the relationship between serum B12 levels and α-Klotho concentrations in US adults and to explore the new insights it provides into the potential mechanisms of aging and related diseases. By clarifying the relationship, our study is expected to provide new ideas to guide clinical practice to improve α-Klotho expression by regulating B12 levels, which may slow down the aging process and reduce the risk of age-related diseases.

## 2 Materials and methods

### 2.1 Source of data

Conducted by the National Centre for Health Statistics (NCHS), NHANES is a program of studies aimed at assessing the health and nutritional status of adults and children in the United States. This survey stands out due to its combination of interviews and physical examinations. Information regarding survey methods, contents, analytic guidelines, and procedure manuals can be accessed at https://www.cdc.gov/nchs/nhanes/ (acquired June 2022). Approval for all NHANES protocols was granted by the NCHS Ethics Review Board, and a consent form was completed by all survey participants.

### 2.2 Study population

**Figure 1:**
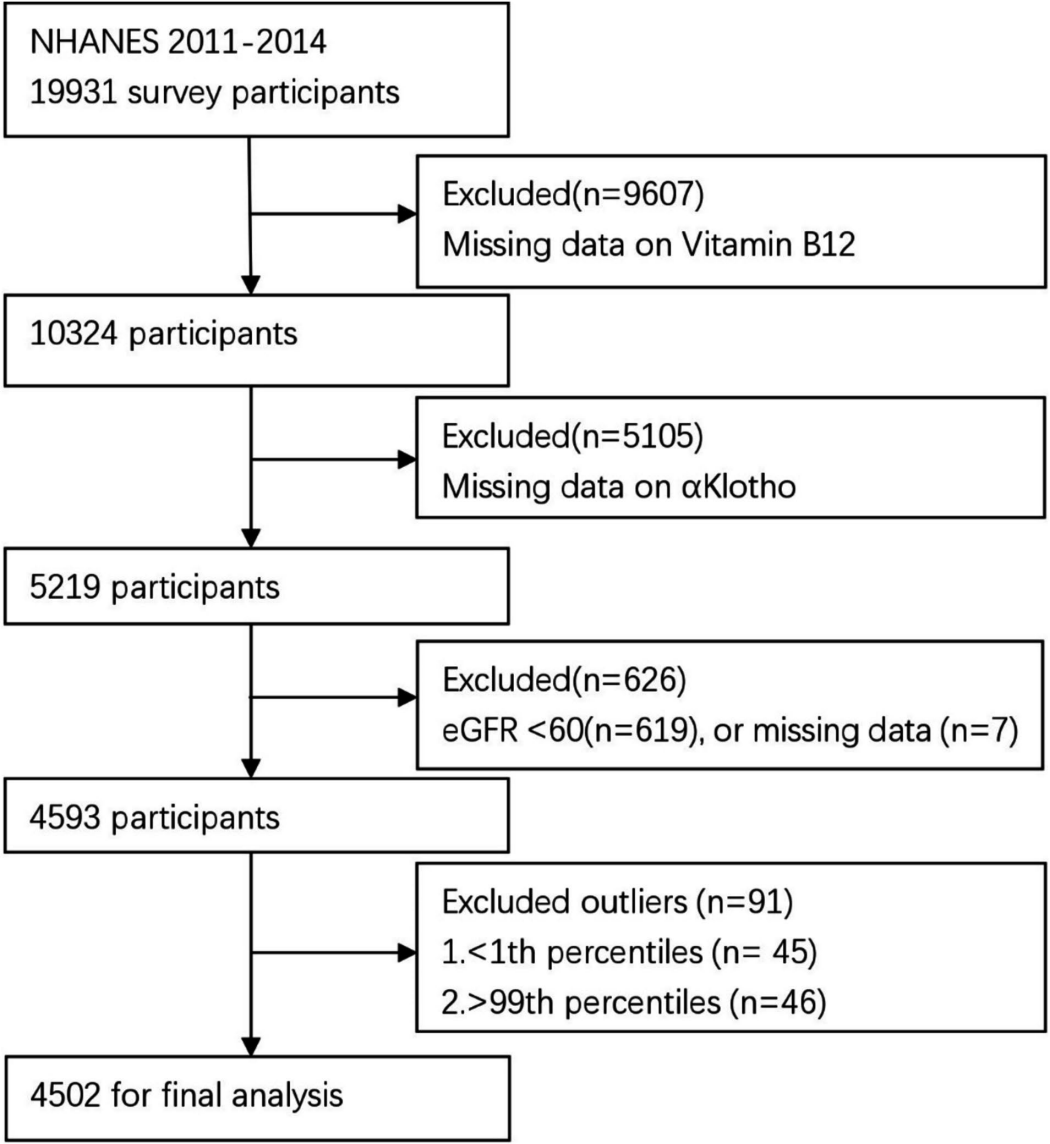
Flow chart of the participant selection process.

The cross-sectional study data were derived from NHANES 2011–2014, encompassing a total of 19,931 participants who were enrolled in the NHANES 2011–2014. Exclusion criteria included: (1) missing B12 data (*n* = 9,607), (2) missing α-Klotho data (*n* = 5,105), (3) eGFR <60 mL/min/1.73/m^2^ (*n* = 619) or missing eGFR data (*n* = 7) (2), and (4) those with B12 outliers (below 1st percentile or above 99th percentile). After exclusion, the final analysis included 4,502 participants (Figure 1).

### 2.3 Exposure and outcome variables

Serum B12 levels, which served as the exposure variable in this research, were assessed through the fully automated electrochemiluminescence immunoassay ‘ECLIA’ and the Bio-Rad Quantiphase II Folate/B12 radio assay. Soluble α-Klotho was the dependent variable. The analyses were conducted utilizing a kit for enzyme-linked immunoadsorption assay that is commercially available from IBL International in Japan.

### 2.4 Covariates

Various covariates based on published research that were thought to be associated with B12 and/or alpha-Klotho were included in this study. The covariates included information on demographics, responses to questionnaires, results from examinations, and data from laboratory tests. Demographic data included gender, age, education, race, and family poverty income ratio (PIR). Questionnaires were employed to assess all lifestyle and health conditions of the respondents. Lifestyle included alcohol use, smoking status, sleep disorders, and physical activity. Health conditions encompassed hypertension, diabetes, weak kidneys, asthma, COPD, thyroid disorders, liver condition, gout, heart disease, and cancer or malignancy. Standardized protocols were followed to measure and record physical examination data, which included measurements of weight, height, and waist circumference. Laboratory data comprised serum urate, HDL, triglyceride, glucose, HbA1c, alanine aminotransferase (ALT), aspartate transaminase (AST), 25-hydroxyvitamin D3, and serum creatinine. Furthermore, the estimated glomerular filtration rate (eGFR) was computed using the Chronic Kidney Disease Epidemiology Collaboration equation (19). Detailed instructions on specimen collection, processing, laboratory method used, quality assurance, and quality control procedures can be accessed on the internet.^1^

### 2.5 Statistical analysis

NHANES uses a complex multistage probability sampling design. Based on the NHANES analytical guidelines, four-year MEC exam sample weights were used for analyses. We evaluated the normality of the data and presented continuous variables as means along with their standard errors. Group comparisons were made using survey-weighted linear regression. Percentages were used to express categorical variables, and groups were compared using survey-weighted Chi-square tests. Weighted multivariate linear regression models were employed to assess the associations between serum B12 and α-Klotho levels. In addition, the correlation between variables was examined through multiple linear regression. Variables exhibiting a VIF greater than 5 were deemed to possess significant multicollinearity, as per the variance inflation factor (VIF). In the final models, covariates were included as potential confounders if they altered the estimates of serum B12 on α-Klotho by more than 10% or if they exhibited a significant association with α-Klotho (20). In the association analyses, B12 was initially examined as a continuous variable and then categorized into four groups (quartiles) to facilitate the interpretation. Different models were established according to the enhanced reporting guidelines for observational epidemiological studies (20). Four models were employed: Model 1, no adjustment; Model 2, adjusted for race, gender, and age; Model 3, adjusted for demographic variables, lifestyle, and health conditions; Model 4, adjusted for all covariates. To address the non-linear relationship between serum B12 and α-Klotho levels, smooth curve fittings and generalized additive models (GAMs) were used. A piecewise linear regression model was applied to determine the threshold effect of B12 values on α-Klotho in terms of the smoothing plot when non-linearity was observed. To determine whether a threshold exists a likelihood ratio test comparing a one-line model with a piecewise regression model was used. When the smoothed curve exhibited a distinct ratio between B12 and α-Klotho, the inflection point was automatically calculated using the recursive method, where the maximum model likelihood was employed (21). Univariate, stratified, and sensitivity analyses were conducted to identify any independent effect between B12 and α-Klotho levels. Furthermore, an interaction test of these factors was conducted.

All analyses were conducted using the following statistical software packages: R 3.4.3^2^ and Empowerstats.^3^ Statistical significance was set at *p* < 0.05 using a two-sided test.

## 3 Results

### 3.1 Baseline characteristics of participants

The research involved a grand total of 4,502 individuals who were 40 years old or older. Table 1 displays the sociodemographic and clinical features of the chosen participants, categorized according to serum B12 quartiles. The participants’ average age was 57.21 ± 0.26 years, with a female majority comprising over half (52.48%). Higher levels of α-Klotho, HDL, AST, ALT, and thyroid problems were more prevalent among participants within the higher serum B12 quartiles. However, individuals in the upper quartiles of serum B12 demonstrated notably reduced levels of BMI, waist circumference, triglyceride, and serum urate in comparison to those in the lower quartiles (*p* < 0.05).

**Table 1:** Weighted demographic characteristics of the participants.

		Vitamin B12,pg/mL				
Characteristic	All	Q1	Q2	Q3	Q4	*p*-value
*N*	4,502	1,120	1,129	1,127	1,126	
α-Klotho pg/mL	872.68 ± 7.65	833.89 ± 10.72	868.06 ± 12.32	876.31 ± 12.73	917.54 ± 12.30	<0.0001
Vitamin B12, pg/mL	590.59 ± 7.61	305.69 ± 2.01	449.44 ± 1.61	607.75 ± 2.06	1056.94 ± 14.60	<0.0001
Age, years	57.21 ± 0.26	57.77 ± 0.48	57.34 ± 0.50	56.79 ± 0.42	56.92 ± 0.49	0.1207
Family PIR	2.64 ± 0.03	2.64 ± 0.07	2.56 ± 0.08	2.71 ± 0.07	2.65 ± 0.06	0.5806
BMI, kg/m^2^	29.46 ± 0.17	30.03 ± 0.33	29.85 ± 0.28	29.05 ± 0.17	28.80 ± 0.31	0.0005
Waist Circumference, cm	101.53 ± 0.4	103.46 ± 0.74	102.51 ± 0.54	100.76 ± 0.46	99.10 ± 0.74	<0.0001
25-hydroxyvitamin D3, nmol/L	63.97 ± 0.41	64.18 ± 1.01	63.93 ± 1.27	62.86 ± 1.00	65.02 ± 1.09	0.7975
HDL, mg/dL	54.06 ± 0.43	52.60 ± 0.84	54.00 ± 0.54	53.63 ± 0.71	56.26 ± 0.88	0.0094
Triglyceride, mg/dL	161.82 ± 3.44	172.23 ± 7.51	165.01 ± 5.32	156.45 ± 4.21	152.55 ± 4.94	0.0099
Glucose, mg/dl	104.67 ± 0.82	106.02 ± 2.21	105.29 ± 1.20	102.92 ± 1.33	104.44 ± 1.39	0.3805
HbA1c, %	5.77 ± 0.02	5.75 ± 0.05	5.76 ± 0.03	5.72 ± 0.04	5.85 ± 0.05	0.2939
Serum urate mg/dL	5.34 ± 0.03	5.47 ± 0.07	5.39 ± 0.05	5.33 ± 0.05	5.13 ± 0.06	0.0001
eGFR mL/min/1.73/m^2^	86.18 ± 0.43	85.52 ± 0.64	86.58 ± 0.69	87.05 ± 0.94	85.46 ± 0.70	0.8452
AST U/L	26.05 ± 0.34	24.72 ± 0.81	24.45 ± 0.49	27.15 ± 0.77	28.14 ± 0.70	0.0007
ALT U/L	25.39 ± 0.33	23.76 ± 0.64	23.97 ± 0.68	26.90 ± 0.82	27.15 ± 0.73	0.0001
Gender (%)						0.9688
Male	2,162(47.52)	534(48.26)	526(46.94)	531(47.98)	571(46.84)	
Female	2,340(52.48)	586(51.74)	603(53.06)	596(52.02)	555(53.16)	
Race (%)						0.1263
Mexican American	535(11.46)	140(13.55)	124(11.12)	124(10.01)	147(11.18)	
Other Hispanic	467(9.86)	107(8.80)	130(12.17)	111(9.85)	119(8.36)	
Non-Hispanic White people	1839(41.68)	441(38.66)	445(39.88)	478(44.76)	475(43.68)	
Non-Hispanic Black people	1,006(22.87)	271(24.78)	252(21.56)	251(22.99)	232(22.12)	
Other Race – Including Multi-Racial	655(14.12)	161(14.21)	178(15.27)	163(12.39)	153(14.66)	
Education (%)						0.2274
Less than 9th grade	483(10.29)	99(8.34)	144(12.61)	108(10.72)	132(9.28)	
9–11th grade (Includes 12th grade with no diploma)	627(14.18)	161(14.87)	182(16.22)	138(12.71)	146(12.69)	
High school graduate/GED or equivalent	973(22.15)	245(22.70)	239(20.83)	252(21.46)	237(23.88)	
Some college or AA degree	1,284(28.56)	347(29.49)	281(25.06)	348(31.46)	308(28.31)	
College graduate or above	1,134(24.81)	267(24.59)	283(25.28)	281(23.65)	303(25.84)	
Physical activity (%)						0.4397
Activity	1,362(30.27)	344(31.08)	345(31.79)	341(30.3)	332(27.56)	
Inactivity	3,137(69.73)	776(68.92)	783(68.21)	784(69.7)	794(72.44)	
Alcohol use (%)						0.5287
Yes	2,968(70.91)	715(68.13)	735(72.01)	747(71.71)	771(71.93)	
No	1,166(29.09)	316(31.87)	292(27.99)	283(28.29)	275(28.07)	
Smoking status (%)						0.7863
Yes	2,100(47.29)	524(47.14)	507(46.87)	545(48.98)	524(46.02)	
No	2,401(52.71)	596(52.86)	621(53.13)	582(51.02)	602(53.98)	
Sleep disorder (%)						0.4859
Yes	552(12.69)	139(12.06)	158(14.22)	141(12.64)	114(11.68)	
No	3,943(87.31)	981(87.94)	969(85.78)	982(87.36)	1,011(88.32)	
Hypertension (%)						0.9218
Yes	2,112(47.39)	532(48.18)	530(46.7)	528(48.03)	522(46.58)	
No	2,385(52.61)	588(51.82)	597(53.30)	598(51.97)	602(53.42)	
Diabetes (%)						0.2575
Yes	573(12.62)	139(11.94)	155(14.71)	135(12.74)	144(10.79)	
No	3,726(87.38)	924(88.06)	917(85.29)	944(87.26)	941(89.21)	
Weak kidneys (%)						0.7888
Yes	183(4.06)	51(4.00)	38(3.40)	46(4.45)	48(4.43)	
No	4,311(95.94)	1,066(96.00)	1,089(96.60)	1,081(95.55)	1,075(95.57)	
Asthma (%)						0.6016
Yes	632(13.57)	159(12.79)	159(13.79)	159(14.85)	155(12.74)	
No	3,866(86.43)	960(87.21)	968(86.21)	968(85.15)	970(87.26)	
Congestive heart failure (%)						0.8986
Yes	164(3.94)	45(3.48)	37(3.86)	37(4.47)	45(3.93)	
No	4,327(96.06)	1,070(96.52)	1,090(96.14)	1,088(95.53)	1,079(96.07)	
Coronary heart disease (%)						0.7138
Yes	197(4.47)	48(3.85)	48(5.13)	49(4.65)	52(4.21)	
No	4,293(95.53)	1,066(96.15)	1,079(94.87)	1,075(95.35)	1,073(95.79)	
COPD (%)						0.418
Yes	304(7.54)	72(6.98)	83(9.24)	79(7.10)	70(6.69)	
No	4,186(92.46)	1,043(93.02)	1,043(90.76)	1,048(92.9)	1,052(93.31)	
Thyroid problem (%)						0.032
Yes	500(12.89)	104(10.34)	106(11.89)	139(13.39)	151(16.39)	
No	3,990(87.11)	1,015(89.66)	1,022(88.11)	984(86.61)	969(83.61)	
Liver condition (%)						0.1259
Yes	218(4.25)	48(2.99)	50(4.26)	58(4.99)	62(4.81)	
No	4,274(95.75)	1,068(97.01)	1,078(95.74)	1,065(95.01)	1,063(95.19)	
Stroke (%)						0.138
Yes	158(2.53)	48(3.11)	33(1.64)	35(2.25)	42(3.27)	
No	4,340(97.47)	1,071(96.89)	1,095(98.36)	1,091(97.75)	1,083(96.73)	
Gout (%)						0.7794
Yes	210(4.87)	56(5.33)	52(5.08)	47(4.13)	55(4.93)	
No	4,289(95.13)	1,064(94.67)	1,077(94.92)	1,078(95.87)	1,070(95.07)	
Cancer or Malignancy (%)						0.9686
Yes	467(12.75)	119(12.43)	116(12.50)	110(12.82)	122(13.32)	
No	4,032(87.25)	1,001(87.57)	1,001(87.50)	1,016(87.18)	1,004(86.68)	

Values are means ± standard errors or *n* (%). For continuous variables: *P*-value was calculated by survey-weighted linear regression. For categorical variables: *P*-value was determined by survey-weighted Chi-square test. Drinking or smoking means drinking at least 12 drinks a year or smoking at least 100 cigarettes in a lifetime, respectively. ALT, alanine aminotransferase; AST, aspartate transaminase; COPD, chronic obstructive pulmonary disease; eGFR, estimated glomerular filtration rate; Family PIR, family poverty income ratio-PIR.

### 3.2 Association of serum B12 with α-Klotho

The findings of the univariate analysis are illustrated in Table 2. These findings indicated positive correlations between the levels of α-Klotho and B12, HDL cholesterol, HbA1c, eGFR, AST, and ALT. However, inverse associations were observed between α-Klotho and BMI, waist circumference, glucose, triglyceride, serum urate, cancer or malignancy, and gout. The association between serum B12 and α-Klotho levels is summarized in Table 3. In the non-adjusted model, a positive correlation was observed between serum B12 and α-Klotho levels (*β* = 0.05, 95% CI: 0.03–0.08, *p* < 0.0001), which was consistent with the fine-tuning model [Model I: adjusted for gender, age, years (Smooth); (*β* = 0.05, 95% CI: 0.03–0.08, *p* < 0.0001)]. After adjusting Model I into Model II [model: model I + Alcohol use, Smoking status, Sleep disorder, Hypertension, Diabetes, Weak kidneys, Asthma, Congestive heart failure, Coronary heart disease, COPD, Thyroid problem, Liver condition, Stroke, Gout, Cancer or Malignancy, BMI (Smooth)], the positive correlation between them remained (*β* = 0.05, 95% CI: 0.02–0.07, *p* = 0.001). In the fully adjusted model III (Adjust model III: model II + 25-hydroxyvitamin D3, HDL, Triglyceride, Glucose, HbA1c, Serum urate, eGFR, AST, ALT), the *β* of α-Klotho was 0.02 (95% CI: 0.00–0.05, *p* = 0.0755). Moreover, a sensitivity analysis was performed, analyzing B12 as a categorical variable (quartile), and the same trend was observed across quartiles (*P* for the trend was <0.0001).

**Table 2:** The results of univariate analysis for α-Klotho, pg/mL.

Covariate	Statistics	Effect size (β)	*P*-value
Vitamin B12, pg/mL	610.37 ± 364.07	0.06 (0.03, 0.08)	<0.0001
Vitamin B12,pg/mL Tertile			
Low	1,500 (33.32%)	Reference	
Middle	1,498 (33.27%)	27.31 (6.01, 48.61)	0.012
High	1,504 (33.41%)	70.77 (49.49, 92.05)	<0.0001
Gender			
Male	2,162 (48.02%)	Reference	
Female	2,340 (51.98%)	−5.64(−23.11,11.84)	0.5272
Age, years	57.30 ± 10.71	0.05 (−0.76, 0.87)	0.9016
Age, years Tertile			
Low	1,422 (31.59%)	Reference	
Middle	1,543 (34.27%)	−9.02 (−30.55, 12.52)	0.4118
High	1,537 (34.14%)	0.38 (−21.18, 21.94)	0.9724
Race/Hispanic origin			
Mexican American	535 (11.88%)	Reference	
Other Hispanic	467 (10.37%)	8.31 (−28.79, 45.41)	0.6607
Non-Hispanic White people	1839 (40.85%)	6.39 (−22.38, 35.17)	0.6633
Non-Hispanic Black people	1,006 (22.35%)	13.20 (−18.15, 44.54)	0.4094
Other Race – Including Multi-Racial	655 (14.55%)	27.98 (−6.16, 62.11)	0.1083
Education			
Less than 9th grade	483 (10.73%)	Reference	
9–11th grade (includes 12th grade with no diploma)	627 (13.93%)	−21.20 (−56.66, 14.26)	0.2413
High school graduate/GED or equivalent	973 (21.62%)	−0.21 (−32.81, 32.39)	0.9901
Some college or AA degree	1,284 (28.53%)	−23.72 (−54.98, 7.54)	0.1371
College graduate or above	1,134 (25.19%)	−8.36 (−40.18, 23.47)	0.6068
Family PIR	2.62 ± 1.67	−0.71 (−6.12, 4.70)	0.7967
Family PIR Tertile			
Low	1,375 (32.97%)	Reference	
Middle	1,400 (33.57%)	4.77 (−17.32, 26.87)	0.6719
High	1,395 (33.45%)	1.44 (−20.68, 23.56)	0.8984
Physical activity			
Activity	1,362 (30.27%)	Reference	
Inactivity	3,137 (69.73%)	−6.22 (−25.23, 12.80)	0.5217
Alcohol use			
Yes	2,968 (71.79%)	Reference	
No	1,166 (28.21%)	−12.53 (−32.80, 7.73)	0.2256
Smoking status			
Yes	2,100 (46.66%)	Reference	
No	2,401 (53.34%)	−1.31 (−18.82, 16.19)	0.8833
Sleep disorder			
Yes	552 (12.28%)	Reference	
No	3,943 (87.72%)	17.43 (−9.20, 44.05)	0.1996
Hypertension			
Yes	2,112 (46.96%)	Reference	
No	2,385 (53.04%)	8.35 (−9.16, 25.86)	0.3500
Diabetes			
Yes	573 (13.33%)	Reference	
No	3,726 (86.67%)	16.18 (−9.99, 42.36)	0.2257
Weak kidneys			
Yes	183 (4.07%)	Reference	
No	4,311 (95.93%)	−43.07 (−87.15, 1.01)	0.0556
Asthma			
Yes	632 (14.05%)	Reference	
No	3,866 (85.95%)	11.07 (−14.07, 36.21)	0.3882
Congestive heart failure			
Yes	164 (3.65%)	Reference	
No	4,327 (96.35%)	−26.73 (−73.27, 19.81)	0.2604
Coronary heart disease			
Yes	197 (4.39%)	Reference	
No	4,293 (95.61%)	9.37 (−33.33, 52.07)	0.6670
COPD			
Yes	304 (6.77%)	Reference	
No	4,186 (93.23%)	34.63 (−0.14, 69.39)	0.0510
Thyroid problem			
Yes	500 (11.14%)	Reference	
No	3,990 (88.86%)	−22.14 (−49.92, 5.65)	0.1184
Liver condition			
Yes	218 (4.85%)	Reference	
No	4,274 (95.15%)	−28.48 (−69.17, 12.21)	0.1702
Stroke			
Yes	158 (3.51%)	Reference	
No	4,340 (96.49%)	30.43 (−17.03, 77.88)	0.2089
Gout			
Yes	210 (4.67%)	Reference	
No	4,289 (95.33%)	46.59 (5.19, 87.98)	0.0274
Cancer or Malignancy			
Yes	467 (10.38%)	Reference	
No	4,032 (89.62%)	37.68 (9.06, 66.30)	0.0099
BMI, kg/m^2^	29.45 ± 6.83	0.09 (−1.20, 1.37)	0.8942
BMI, kg/m^2^ Tertile			
Low	1,464 (32.83%)	Reference	
Middle	1,508 (33.81%)	−31.04 (−52.51, −9.58)	0.0046
High	1,488 (33.36%)	−11.56 (−33.09, 9.98)	0.2929
Waist circumference, cm	100.79 ± 15.53	−0.84 (−1.41, −0.27)	0.0041
Waist Circumference, cm Tertile			
Low	1,431 (33.03%)	Reference	
Middle	1,452 (33.52%)	−21.16 (−42.96, 0.63)	0.0571
High	1,449 (33.45%)	−25.80 (−47.61, −3.99)	0.0205
25-hydroxyvitamin D3,nmol/L	63.69 ± 28.89	0.06 (−0.25, 0.36)	0.7198
25-hydroxyvitamin D3,nmol/L Tertile			
Low	1,501 (33.34%)	Reference	
Middle	1,500 (33.32%)	11.97 (−9.41, 33.36)	0.2726
High	1,501 (33.34%)	6.82 (−14.57, 28.20)	0.5320
HDL, mg/dl	53.41 ± 15.92	0.28 (−0.26, 0.83)	0.3104
HDL, mg/dl Tertile			
Low	1,405 (31.21%)	Reference	
Middle	1,522 (33.81%)	22.43 (0.77, 44.09)	0.0425
High	1,575 (34.98%)	25.15 (3.67, 46.64)	0.0218
Triglyceride, mg/dl	161.73 ± 151.96	−0.08 (−0.13, −0.02)	0.0103
Triglyceride, mg/dl Tertile			
Low	1,482 (32.95%)	Reference	
Middle	1,507 (33.50%)	−25.81 (−47.21, −4.40)	0.0182
High	1,509 (33.55%)	−39.91 (−61.31, −18.52)	0.0003
Glucose, mg/dl	108.29 ± 45.41	0.65 (0.46, 0.85)	<0.0001
Glucose, mg/dl Tertile			
Low	1,447 (32.14%)	Reference	
Middle	1,554 (34.52%)	−19.44 (−40.82, 1.94)	0.0748
High	1,501 (33.34%)	14.37 (−7.20, 35.93)	0.1917
HbA1c,%	5.92 ± 1.19	19.87 (12.54, 27.20)	<0.0001
HbA1c,% Tertile			
Low	1,127 (25.04%)	Reference	
Middle	1800 (40.00%)	−9.71 (−31.95, 12.53)	0.3922
High	1,573 (34.96%)	15.42 (−7.44, 38.27)	0.1862
Serum urate, mg/dL	5.36 ± 1.34	−27.11 (−33.59, −20.64)	<0.0001
Serum urate, mg/dL Tertile			
Low	1,422 (31.60%)	Reference	
Middle	1,557 (34.60%)	−25.65 (−47.02, −4.27)	0.0187
High	1,521 (33.80%)	−75.78 (−97.27, −54.28)	<0.0001
eGFR, mL/min/1.73/m^2^	88.61 ± 20.76	1.10 (0.68, 1.52)	<0.0001
eGFR, mL/min/1.73/m^2^ Tertile			
Low	1,501 (33.34%)	Reference	
Middle	1,500 (33.32%)	24.96 (3.62, 46.29)	0.0219
High	1,501 (33.34%)	51.57 (30.23, 72.90)	<0.0001
AST, U/L	25.86 ± 15.58	1.23 (0.67, 1.79)	<0.0001
AST, U/L Tertile			
Low	1,468 (32.64%)	Reference	
Middle	1,503 (33.41%)	11.26 (−10.21, 32.73)	0.3040
High	1,527 (33.95%)	38.43 (17.04, 59.82)	0.0004
ALT, U/L	24.92 ± 16.49	0.68 (0.15, 1.21)	0.0120
ALT, U/L Tertile			
Low	1,368 (30.42%)	Reference	
Middle	1,564 (34.78%)	−16.79 (−38.47, 4.90)	0.1293
High	1,565 (34.80%)	5.68 (−16.00, 27.36)	0.6075

β (95%CI). Drinking or smoking means drinking at least 12 drinks a year or smoking at least 100 cigarettes in a lifetime, respectively. ALT, alanine aminotransferase; AST, aspartate transaminase; COPD, chronic obstructive pulmonary disease; eGFR, estimated glomerular filtration rate; Family PIR, family poverty income ratio-PIR.

**Table 3:** Association between serum vitamin B12 level (pg/mL) and serum α-klotho (pg/mL) levels.

	Non-adjusted model		Model I		Model II		Model III	
	*β* (95%CI)	*P*-value	*β* (95%CI)	*P*-value	*β* (95%CI)	*P*-value	*β* (95%CI)	*P*-value
Vitamin B12	0.05 (0.03, 0.08)	<0.0001	0.05 (0.03, 0.08)	<0.0001	0.05 (0.02, 0.07)	0.001	0.02 (−0.00, 0.05)	0.0755
Vitamin B12 quartile								
Q1	Reference		Reference		Reference		Reference	
Q2	34.17 (11.15, 57.18)	0.0036	34.61 (11.53, 57.69)	0.0033	46.05 (20.62, 71.48)	0.0004	48.71 (23.70, 73.73)	0.0001
Q3	42.42 (19.25, 65.59)	0.0003	43.09 (19.82, 66.36)	0.0003	53.36 (28.11, 78.61)	<0.0001	49.14 (24.02, 74.26)	0.0001
Q4	83.65 (59.67, 107.62)	<0.0001	84.27 (60.22, 108.31)	<0.0001	83.98 (57.98, 109.97)	<0.0001	68.90 (42.81, 94.99)	<0.0001
*P* for trend	<0.0001		<0.0001		<0.0001		<0.0001	

Adjust I model was adjusted for: gender; age, years (smooth); race. Adjust II model was adjusted for: model I + alcohol use; smoking status; sleep disorder; hypertension; diabetes; weak kidneys; asthma; congestive heart failure; coronary heart disease; COPD; thyroid problem; liver condition; stroke; gout; cancer or malignancy; BMI, kg/m^2^ (Smooth). Generalized additive models were applied. Adjust III model was adjusted for: model II + 25-hydroxyvitamin D3 (smooth); HDL (smooth); triglyceride (smooth); glucose (smooth); HbA1c (smooth); serum urate; eGFR (smooth); AST (smooth); ALT (smooth).

### 3.3 Non-linear association of serum B12 with α-Klotho

**Figure 2:**
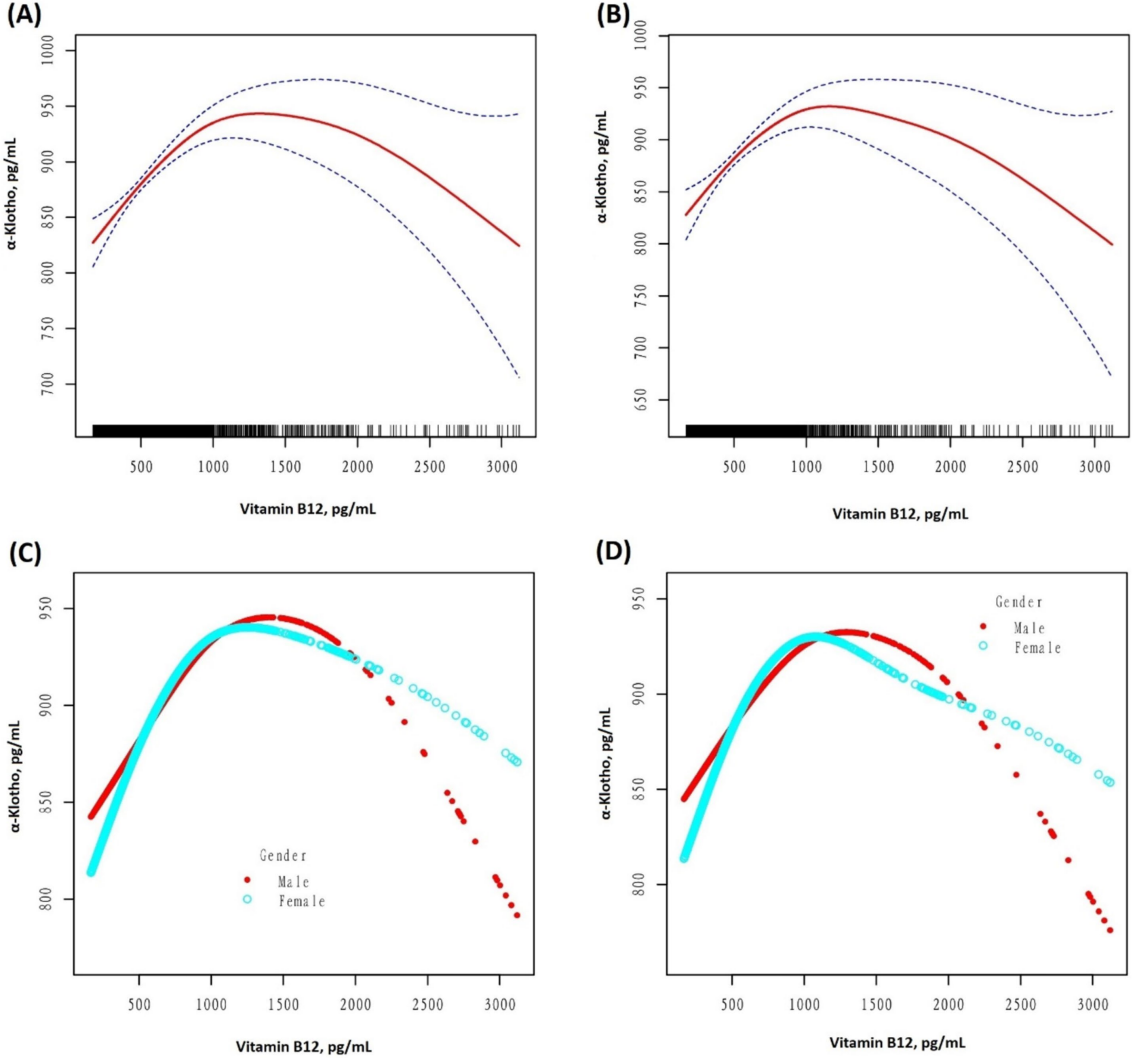
**(A)** A threshold, a non-linear association between the serum vitamin B12 (pg/mL) and serum α-Klotho (pg/mL) levels was observed in the unadjusted model **(A,C)** (*p* < 0.001, 0.0023) and adjusted model **(B,D)** (*p* < 0.001, <0.001), in a generalized additive model. In graphs **(A,B)**, the red and blue lines represent the smoothed curve fits between the variables and their 95% confidence intervals, respectively. All adjusted for Age; Race/Hispanic origin; Alcohol use; Smoking status; Sleep disorder; Hypertension; Diabetes; Weak kidneys; Asthma; Congestive heart failure; Coronary heart disease; COPD; Thyroid problem; Liver condition; Stroke; Gout; Cancer or Malignancy; BMI; 25-hydroxyvitamin D3; HDL; Triglyceride; Glucose; HbA1c; Serum urate; eGFR; AST; ALT.

After unadjusting and adjusting for other covariables, an inverted U-shaped curve association between serum B12 and α-Klotho levels was observed in the GAM, as illustrated in Figure 2. Moreover, when stratified by sex, a similar inverted U-shaped curve association was observed between men and women. The curve inflection points of B12 in the total population, males, and females were 1020.00, 1030.00, and 921.00 pg/mL, respectively, as indicated in Table 4. To calculate the saturation effect of B12 on α-Klotho based on the smoothing curve and its inflection point, a two-stage logistic regression model was employed. This analysis revealed a positive correlation between B12 and α-Klotho on the left side (total: B12 < 1020.00 pg/mL; male <1030.00 pg/mL; female <921.00 pg/mL) of the inflection point (total: *β* = 0.14, 95% CI: 0.09–0.18, *p* < 0.0001; male: *β* = 0.14, 95% CI: 0.07–0.20, *p* < 0.0001; female: *β* = 0.15, 95% CI: 0.80–0.21, *p* < 0.0001). Conversely, on the right side (total: B12 > 1020.00 pg/mL; male >1030.00 pg/mL; female >921.00 pg/mL) of the inflection point, a negative correlation between B12 and α-Klotho was observed (total: *β* = −0.12, 95% CI:−0.17−−0.06, *p* < 0.0001; male: *β* = −0.11, 95% CI:−0.19−−0.03, *p* = 0.0069; female: *β* = −0.11, 95% CI:−0.18−−0.04, *p* = 0.0015) (Table 4).

**Table 4:** Threshold effect analysis of serum vitamin B12 (pg/mL) and serum α-Klotho (pg/mL) levels using piece-wise linear regression.

Models	Total		Male		Female	
	*β* (95%CI)	*P*-value	*β* (95%CI)	*P*-value	*β* (95%CI)	*P*-value
Model I						
One line effect	0.03 (0.01, 0.06)	0.0195	0.04 (−0.00, 0.08)	0.0749	0.02 (−0.01, 0.06)	0.1919
Model II						
Inflection point (K)	1020.00		1030.00		921.00	
<*K*	0.14 (0.09, 0.18)	<0.0001	0.14 (0.07, 0.20)	<0.0001	0.15 (0.08, 0.21)	<0.0001
>*K*	−0.12 (−0.17, −0.06)	<0.0001	−0.11 (−0.19, −0.03)	0.0069	−0.11 (−0.18, −0.04)	0.0015
Model fit value at *K*	948.94 (926.50, 971.38)		954.83 (921.55, 988.11)		939.77 (912.11, 967.43)	
LRT test	<0.001		<0.001		<0.001	

Models I and II represent one-dimensional linear regression and two-stage regression, respectively. Adjusted for: age; race; alcohol use; smoking status; sleep disorder; hypertension; diabetes; weak kidneys; asthma; congestive heart failure; coronary heart disease; COPD; thyroid problem; liver condition; stroke; gout; cancer or malignancy; BMI; 25-hydroxyvitamin D3; HDL; triglyceride; glucose; HbA1c; serum urate; eGFR; AST; ALT.

### 3.4 Subgroup analyses

**Figure 3:**
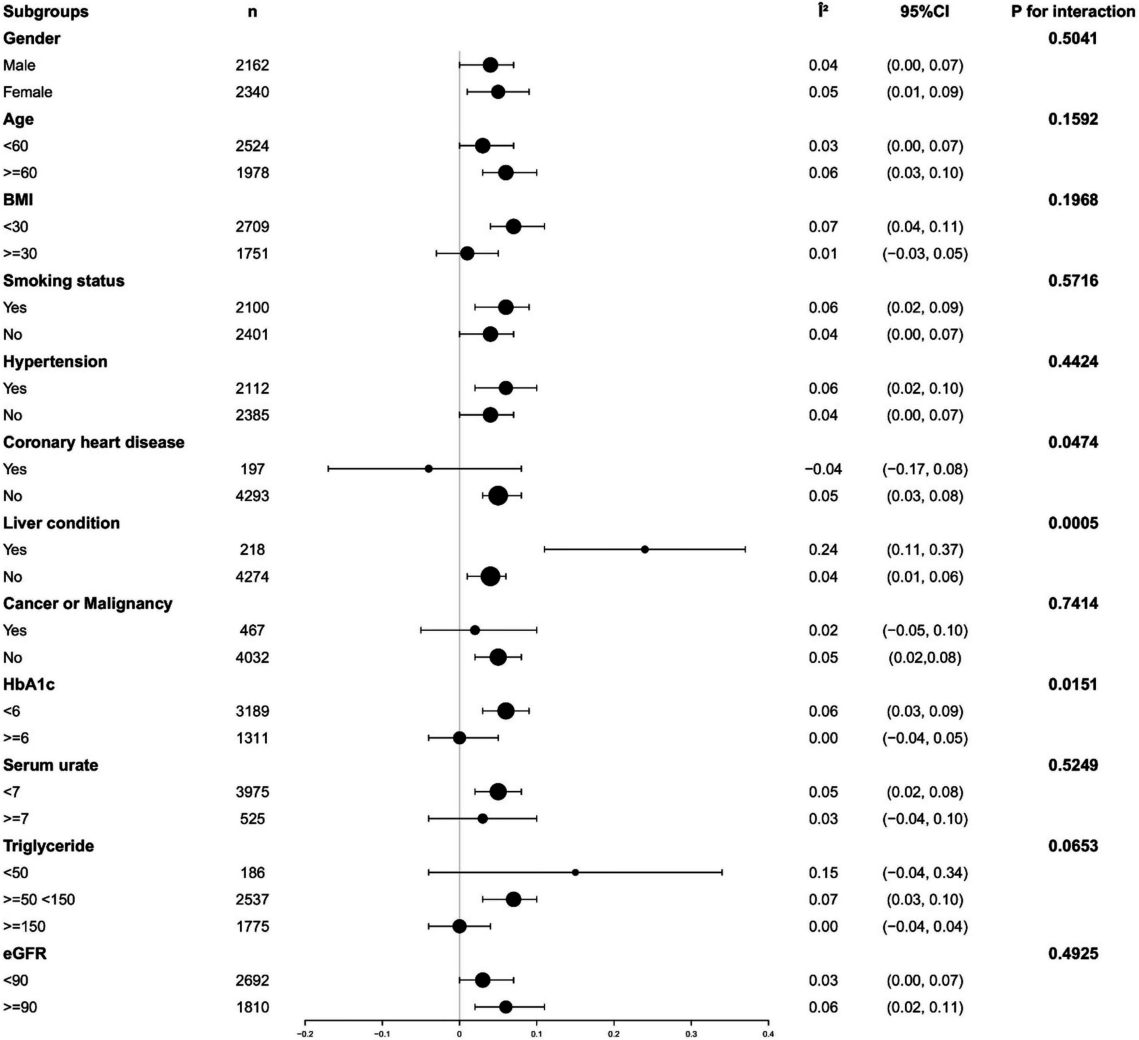
Analyzing subgroups to examine the correlation between levels of serum vitamin B12 and serum α-Klotho. Adjusted for gender, age, race, alcohol use, smoking status, sleep disorder, hypertension, diabetes, weak kidneys, asthma, Congestive heart failure; Coronary heart disease, COPD, Thyroid problem, Liver condition, Stroke, Gout, Cancer or Malignancy, BMI, 25-hydroxyvitamin D3, HDL, Triglyceride, Glucose, HbA1c, Serum urate, eGFR, AST, ALT except the subgroup variable.

Analyses were performed by dividing participants into subgroups based on major covariates known to affect B12 and α-Klotho levels. Among participants with coronary heart disease, high triglycerides, or high glycosylated hemoglobin, plasma B12 exhibited a significantly weakened positive association with α-Klotho. However, this positive correlation was considerably enhanced in participants with liver disease (all *p-*values for interaction <0.05). No statistically significant interactions were observed for gender, age, BMI, serum urate, eGFR, smoking status, hypertension, stroke, and cancer or malignancy (all *p*-values for interaction >0.05). Detailed findings are illustrated in Figure 3. To verify the effect of oral administration of B12-containing medications or dietary supplements on the primary outcome, we aimed to assess their impact. A total of 1,542 out of 4,502 participants were excluded because they had taken B12-containing medications or dietary supplements. The remaining 2,960 participants were analyzed as a subgroup of the study population. The results of the subgroup analyses showed general agreement with the core results of the total study population. Detailed findings are illustrated in Supplementary Tables S3, S4.

### 3.5 Sensitivity analyses

Supplementary Tables S1, S2 and Supplementary Figure S1 show the results of the sensitivity analysis. In the sensitivity analysis, the population under 60 years of age and the non-Hispanic White population were selected for separate analysis, which was consistent with the overall results. This indicates that the results of this study exhibit good stability in US Adults.

## 4 Discussion

There was a significant inverted U-shaped correlation between B12 and α-Klotho, and this correlation was independent of other risk factors, which was different from previous studies (18). It is speculated that it may be related to the differences in the selected population and adjustment variables. In this study, not only was the independent impact of B12 on α-Klotho concentrations evaluated but also a non-linear relationship between B12 and α-Klotho concentrations was identified for the first time, with the inflection point of B12 computed as (1020.00 pg/mL). This association between B12 and α-klotho exerted contrasting effects on the left and right sides of the inflection point. This finding demonstrates that excessively low or high B12 inhibits α-Klotho expression.

The results of this study have important clinical significance for guiding clinicians in the rational use of B12. The inverted U-shaped relationship suggests that clinicians should promptly initiate B12 supplementation upon detecting B12 deficiency, while also paying attention to the detection of B12 levels to prevent excessive supplementation. In cases of identified B12 excess, if attributed to excessive intake, clinicians should reduce the intake. Simultaneously, attention should also be directed toward potential underlying diseases that could cause B12 abnormalities. When the serum B12 concentrations in men and women were 1030.00 and 921.00 pg/mL, respectively, the concentration of α-klotho was also the highest. Thus, for the first time, we propose that these two inflection points are the optimal B12 levels in men and women. Clinicians support α-Klotho expression and maintain overall health by maintaining human B12 levels at optimal levels. However, the mechanism underlying this inverted U-shaped curvilinear relationship remains unclear.

**Figure 4:**
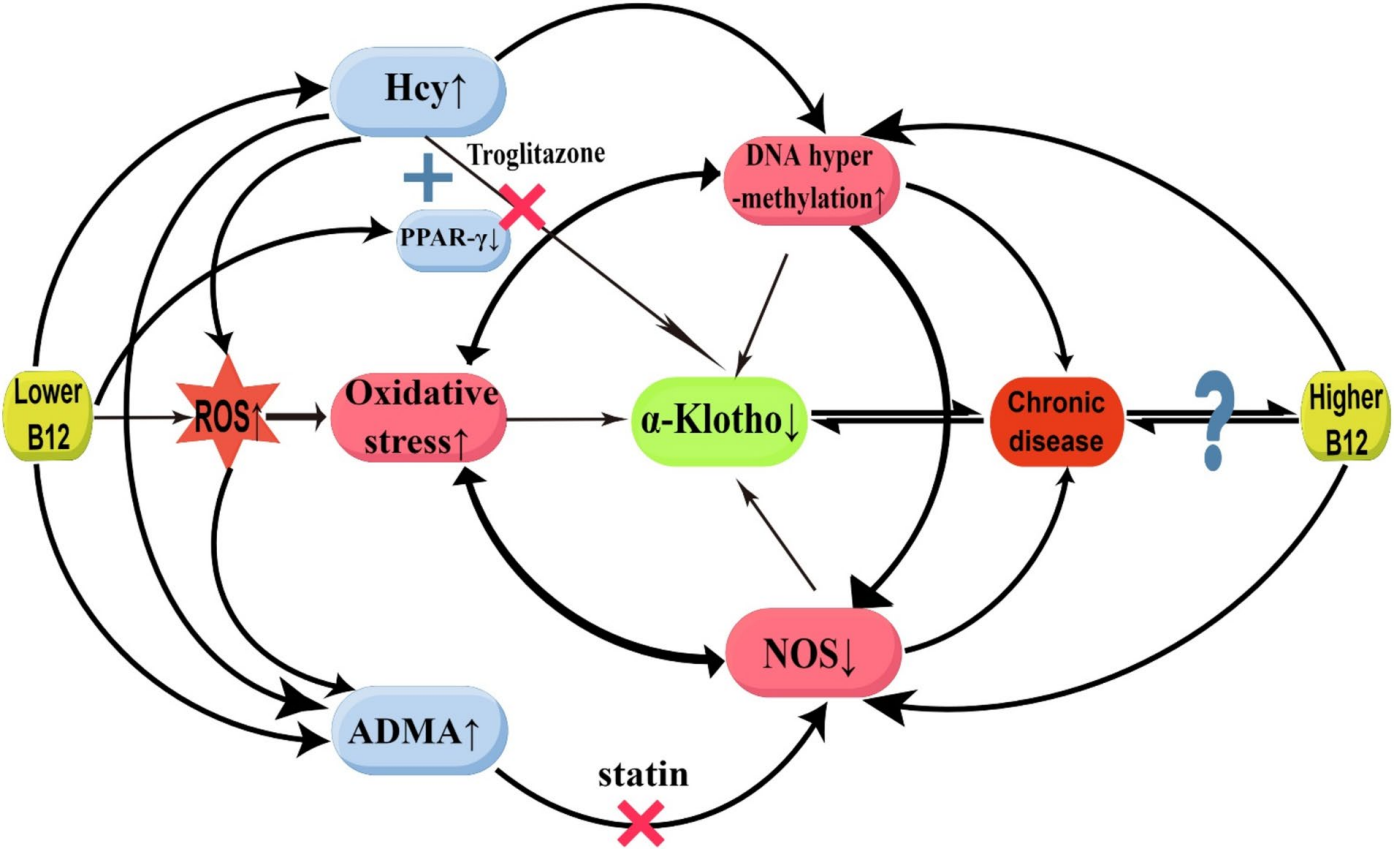
Possible mechanisms for the relationship between vitamin B12 and α-Klotho. ADMA, asymmetric dimethylarginine; B12, Vitamin B12; Hcy, homocysteinemia; NOS, oxide synthase; PPAR*γ*, peroxisome proliferator-activated receptor γ; ROS, reactive oxygen species. ↑: enhancement; ↓: suppression; ×: block (This figure was drawn by Figdraw.com).

In our study, a positive correlation between α-Klotho and B12 concentrations was observed when serum B12 levels were below (1020.00 pg/mL). In previous study, it was discovered that B12 deficiency can result in elevated serum homocysteine (Hcy) and asymmetric dimethylarginine (ADMA), whereas Hcy and oxidative stress were found to increase ADMA accumulation by inhibiting ADAM-degrading enzyme expression (22, 23). However, B12 supplementation was found to reduce Hcy levels (24). The potential mechanisms through which B12 deficiency induces a decrease in serum α-Klotho are hypothesized as follows: first, Hcy inhibits Klotho production by causing ROS accumulation, thereby triggering oxidative stress and activating inflammatory pathways (25). Hcy may produce hydrogen peroxide and superoxide anion through disulfide bond formation, which increases the oxidative degradation of nitric oxide (NO), resulting in oxidative stress (22). Furthermore, HHCY directly inhibits α-klotho expression by binding to peroxisome proliferator-activated receptor *γ* (PPARγ). Klotho has been identified as a direct downstream target of PPARγ (26), and studies have determined that PPARγ upregulates α-Klotho gene expression (26, 27). PPARγ activation can enhance Klotho expression, thus protecting kidney function (28). The PPAR-γ agonist, troglitazone, binds with PPAR-γ, thus enhancing Klotho gene expression in the kidneys (29). Hcy can compete with PPAR-γ agonists for binding to PPAR-γ receptors, counteracting the effect of PPAR-γ agonists in increasing Klotho gene expression (30). Furthermore, B12 deficiency down-regulates PPARγ gene expression (31, 32). The reduced PPARγ gene expression further undermines its capacity to up-regulate α-Klotho. Second, B12 directly scavenges ROS, particularly superoxide, thereby counteracting oxidative stress (14). Superoxide production is increased in B12 deficiency, thereby inducing oxidative stress and inflammatory responses and inhibiting α-Klotho expression (7). Third, ADMA serves as an endogenous nitric oxide synthase (NOS) inhibitor, and B12 deficiency-elevated ADMA can inhibit α-Klotho expression by inhibiting NOS production, resulting in reduced NO synthesis and oxidative stress (22, 23). Research has demonstrated that chronic NOS blockade reduces renal Klotho protein expression; in NOS-blocked rats, chronic statin treatment restores Klotho expression (33). Through the mechanisms elucidated above, we hypothesized that B12 restores α-Klotho expression as B12 levels in the body gradually increase from a deficient state (Figure 4).

In our study, a negative correlation between α-Klotho and B12 concentrations was observed when the serum B12 level exceeded (1020.00 pg/mL). A previous study demonstrated a significant reduction in plasma Hcy levels among patients subjected to prolonged high-dose B12 treatment; however, an upsurge in cardiovascular incidents and a dramatic deterioration in kidney function were observed (34). Moreover, high B12 concentrations are accompanied by high mortality rates (35). This is similar to our results. It has been hypothesized that excessive B12 may inhibit α-Klotho expression through the following mechanisms: first, excessive B12 is accompanied by DNA hypermethylation (34, 36), and Klotho promoter hypermethylation inhibits α-Klotho expression (6). Several studies have further shown that elevated DNA methyltransferases lead to hypermethylation of the Klotho promoter, which results in decreased α-Klotho levels. Conversely, inhibiting the elevation of DNA methyltransferases can normalize promoter DNA hypermethylation and restore α-Klotho expressions (37, 38). Klotho levels in both the renal and serum can be increased by activating DNA demethylation enzymes (39). In cases of moderate B12 concentration, it is likely to impact the expression level of the Klotho gene by regulating its methylation status. However, excessively high B12 concentrations may result in DNA hypermethylation, thereby suppressing Klotho gene expression. Furthermore, excessive endogenous cobalamin *in vivo* could modulate NOS activity, indirectly downregulating α-Klotho expression under both normal and pathological conditions. Some investigations have identified direct interactions between cobalamin and NOS, which significantly inhibit NOS activity (40, 41). Moreover, research has identified high serum B12 levels among patients with various conditions, including alcoholism, liver disease, hematologic disorders, cancer, kidney disease, autoimmune disease, and bronchopulmonary disease (42). Whether B12 serves as a marker or a direct cause of disease remains unclear, and it is speculated that high B12 levels could trigger or result from numerous diseases, and the two interact with each other (Figure 4).

In summary, the lack of B12 can result in the occurrence of oxidative stress (43), inhibit NOS expression, and decrease NO synthesis (44), thereby exacerbating oxidative stress. Oxidative stress-induced inflammation can lead to aberrant DNA methylation, which impairs gene expression and repair. The hypermethylation of NOS promoter can result in decreased NOS expression and NO production disorder (45). Furthermore, HHCY can lead to DNA methylation or hypermethylation through epigenetic mechanisms. Oxidative stress, abnormal DNA methylation, and decreased NOS expression establish a positive feedback loop that strengthens the inhibition of α-Klotho expression. Additionally, excessive B12 participates in the above positive feedback loop through DNA hypermethylation and direct inhibition of NOS to inhibit α-Klotho expression (45) (Figure 4).

Studies have found that there are differences in B12 levels between different races in the American population, with non-Hispanic Black population showing the highest levels of B12; these variations may be attributed to metabolic differences between races (46). Furthermore, the combination of genetic and acquired/environmental factors may lead to racial differences in serum B12 (47). Individual health status and B12 levels are also influenced by personal and demographic factors. Thus, the results of the study may be potentially influenced by different races and cultures.

Subgroup analyses revealed interactions between α-Klotho and B12 in the context of coronary heart disease, high triglycerides, and high glycosylated hemoglobin. Research has indicated that B12 deficiency can induce the generation of advanced glycosylation end products (AGEs) through oxidative stress. These AGEs can hinder the cellular uptake of B12, thereby establishing a positive feedback loop where oxidative stress-induced peroxidation of carbohydrates and lipids contributes to the development of type 2 diabetes and coronary artery disease and inhibits α-Klotho expression (43, 48). Treatment of B12 deficiency reduces moderate-to-severe HHcy and its potential impact on cardiovascular disease (49). However, pre-existing cardiovascular disease may diminish the beneficial effects of B12 (49, 50). Furthermore, a lack of B12 is linked to elevated levels of hypertriglycerides (51). A previous study highlighted a negative association between hypertriglyceridemia and α-Klotho expression (52). Interestingly, subgroup analyses further revealed that patients with liver disease played an enhancing role, and previous studies have also found α-Klotho to be elevated in patients with cirrhosis and non-alcoholic fatty liver disease-related fibrosis (53, 54). The question of how to explain this counterintuitive result arises. Given the fact that Klotho possesses antioxidant, anti-inflammatory, and antifibrotic characteristics, the rise of α-Klotho in this particular set of individuals could indicate an effort to regulate the escalation of inflammation. While the precise underlying mechanism remains uncertain, the increase in α-Klotho could potentially serve as a compensatory (53).

To the best of our knowledge, this study is the first to propose an inverted U-shaped correlation between serum B12 and serum α-Klotho levels in a large population of various ethnicities. This unique association remained statistically significant, even after adjusting for potential confounding variables. However, several limitations are associated with this study. First, due to its cross-sectional design, establishing a clear causal relationship between serum B12 and serum α-Klotho levels was not feasible. Longitudinal studies are needed to explore the temporal relationship and verify causality. Second, the identification of B12 deficiency solely relied on plasma B12 concentrations, lacking validation through metabolite (plasma Hcy or methylmalonic acid) measurements, which could not provide more comprehensive insights into B12 status. Third, the absence of intermediates such as Hcy and ADMA hindered the elucidation of potential linkage mechanisms. Fourth, patients with chronic kidney disease will show a significant decrease in renal klotho expression, resulting in a significant decrease in serum α-Klotho levels (2, 10). Our study excluded individuals with eGFR <60 mL/min/1.73/m^2^. Therefore, our conclusions cannot be extrapolated to the population with eGFR <60 mL/min/1.73/m^2^. This limitation should be considered when interpreting our findings. Fifth, subgroup analyses indicated that the results were generally consistent between those subgroups that excluded participants taking B12-containing dietary supplements or medications and the results from the total study population. Including participants who took B12-containing dietary supplements or medications would not have altered our primary conclusions. However, the NHANES database did not fully capture data on some other foods or medications, preventing us from determining additional effects, which represents another limitation of our study.

## 5 Conclusion

In conclusion, our results indicate a noteworthy inverted U-shaped association between serum B12 and serum α-Klotho levels in the general population. The findings of this study demonstrate that the decrease in α-Klotho levels may be associated with either excessively high or low B12 concentrations. Based on these findings, we recommend that human serum B12 should be maintained at the optimal concentration. Furthermore, ethical issues should be considered in clinical research programs due to the potential risks of excessive supplementation of B12 and its adverse effects on health outcomes. Our findings will contribute to the rational application of B12 to achieve high α-Klotho concentration and meet the needs of maintaining health and delaying aging. Potential avenues for future research include investigating the effects of B12 supplementation, conducting detailed mechanism studies, and investigating the clinical effects of α-Klotho inhibition associated with B12 levels.

## Data Availability

The datasets presented in this study can be found in online repositories. The names of the repository/repositories and accession number(s) can be found: https://www.cdc.gov/nchs/nhanes/.

## Footnotes

^1^ https://www.cdc.gov/nchs/nhanes/

^2^ http://www.R-project.org

^3^ http://www.empowerstats.com
