# Supplementary material for: Inverted U-shaped relationship between serum vitamin B12 and α-Klotho levels in US adults: a cross-sectional study
Source: Front Nutr. 2024 Oct 23;11:1473196. doi: 10.3389/fnut.2024.1473196 (PMC11539862; doi:10.3389/fnut.2024.1473196)
Supplement: Supplementary file 1 [file Data_Sheet_1.docx]

Supplementary Table 1 Sensitivity analysis.(Age<60, years)

|  | Non-adjusted model(n=2524) | |  | Model Ⅰ(n=2091) | |  | Model Ⅱ(n=2091) | |  |
| --- | --- | --- | --- | --- | --- | --- | --- | --- | --- |
|  | β (95%CI) | *P*-value |  | β (95%CI) | *P*-value |  | β (95%CI) | *P*-value |  |
| Vitamin B12 | 0.05 (0.02, 0.08) | 0.0015 |  | 0.05 (0.02, 0.08) | 0.0016 |  | 0.02 (-0.02, 0.05) | 0.3777 |  |
| Vitamin B12 quartile |  |  |  |  |  |  |  |  |  |
| Q1 | Reference |  |  | Reference |  |  | Reference |  |  |
| Q2 | 27.26 (-3.03, 57.54) | 0.0779 |  | 27.12 (-3.28, 57.52) | 0.0805 |  | 40.85 (7.90, 73.79) | 0.0152 |  |
| Q3 | 29.75 (-0.64, 60.15) | 0.0551 |  | 29.37 (-1.27, 60.01) | 0.0604 |  | 27.90 (-5.84, 61.28) | 0.1016 |  |
| Q4 | 73.72 (42.22, 105.21) | <0.0001 |  | 73.74 (42.10, 105.38) | <0.0001 |  | 48.11 (13.84, 82.38) | 0.006 |  |
| P for trend | <0.0001 |  |  | <0.0001 |  |  | <0.0001 |  |  |

AdjustⅠmodel was adjusted for: Gender; Age, years (Smooth); Race. Adjust Ⅱmodel was adjusted for: model I + Alcohol use; Smoking status; Sleep disorder; Hypertension; Diabetes; Weak kidneys; Asthma; Congestive heart failure; Coronary heart disease; COPD; Thyroid problem; Liver condition; Stroke; Gout; Cancer or Malignancy; BMI, kg/m² (Smooth) ; 25-hydroxyvitamin D3 (Smooth); HDL (Smooth); Triglyceride (Smooth); Glucose (Smooth); HbA1c(Smooth); Serum urate; eGFR (Smooth); AST (Smooth); ALT (Smooth). Generalized additive models were applied.

Supplementary Table 2 Sensitivity analysis.( Non-Hispanic White)

|  | Non-adjusted model(n=1839) | |  | Model Ⅰ(n=1839) | |  | Model Ⅱ(n=1621) | |  |
| --- | --- | --- | --- | --- | --- | --- | --- | --- | --- |
|  | β (95%CI) | *P*-value |  | β (95%CI) | *P*-value |  | β (95%CI) | *P*-value |  |
| Vitamin B12 | 0.04 (0.00, 0.08) | 0.0456 |  | 0.04 (0.00, 0.08) | 0.0414 |  | 0.01 (-0.03, 0.05) | 0.6999 |  |
| Vitamin B12 quartile |  |  |  |  |  |  |  |  |  |
| Q1 | Reference |  |  | Reference |  |  | Reference |  |  |
| Q2 | 35.92 (-0.93, 72.78) | 0.0562 |  | 37.06 (0.22, 73.90) | 0.0488 |  | 53.22 (13.56, 92.88) | 0.0086 |  |
| Q3 | 30.87 (-5.23, 66.97) | 0.0939 |  | 33.65 (-2.52, 69.82) | 0.0684 |  | 45.98 (7.08, 84.88) | 0.0206 |  |
| Q4 | 63.09 (25.59, 100.58) | 0.0010 |  | 64.31 (26.80, 101.82) | 0.0008 |  | 44.45 (3.95, 84.95) | 0.0316 |  |
| P for trend | <0.0001 |  |  | <0.0001 |  |  | <0.0001 |  |  |

AdjustⅠmodel was adjusted for: Gender; Age, years (Smooth). Adjust Ⅱmodel was adjusted for: model I + Alcohol use; Smoking status; Sleep disorder; Hypertension; Diabetes; Weak kidneys; Asthma; Congestive heart failure; Coronary heart disease; COPD; Thyroid problem; Liver condition; Stroke; Gout; Cancer or Malignancy; BMI, kg/m² (Smooth) ; 25-hydroxyvitamin D3 (Smooth); HDL (Smooth); Triglyceride (Smooth); Glucose (Smooth); HbA1c(Smooth); Serum urate; eGFR (Smooth); AST (Smooth); ALT (Smooth).Generalized additive models were applied.

Supplementary Table 3 Association between serum vitamin B12 level (pg/mL) and serum α-klotho(pg/mL) levels. ( Non-vitamin B12 supplementation group)

|  | Non-adjusted model(n=2960) | |  | Model Ⅰ(n=2960) | |  | Model Ⅱ(n=2486) | |  |
| --- | --- | --- | --- | --- | --- | --- | --- | --- | --- |
|  | β (95%CI) | *P*-value |  | β (95%CI) | *P*-value |  | β (95%CI) | *P*-value |  |
| Vitamin B12 | 0.05 (0.02, 0.08) | 0.0035 |  | 0.05 (0.02, 0.08) | 0.0036 |  | 0.01 (-0.03, 0.04) | 0.6015 |  |
| Vitamin B12 quartile |  |  |  |  |  |  |  |  |  |
| Q1 | Reference |  |  | Reference |  |  | Reference |  |  |
| Q2 | 49.73 (20.87, 78.59) | 0.0007 |  | 49.50 (20.57, 78.44) | 0.0008 |  | 63.45 (32.24, 94.67) | <0.0001 |  |
| Q3 | 52.34 (23.33, 81.34) | 0.0004 |  | 51.76 (22.67, 80.85) | 0.0005 |  | 52.90 (21.36, 84.45) | 0.0010 |  |
| Q4 | 90.57 (60.01, 121.14) | <0.0001 |  | 90.36 (59.74, 120.98) | <0.0001 |  | 69.72 (36.49, 103.04) | <0.0001 |  |
| P for trend | <0.0001 |  |  | <0.0001 |  |  | <0.0001 |  |  |

Non-adjusted model adjust for: None 
Adjust I model adjust for: Gender; Age,years; Race/Hispanic origin 
Adjust II model adjust for: Gender; Age,years; Race/Hispanic origin; Alcohol use; Smoking status; Sleep disorder; Hypertension; Diabetes; Weak kidneys; Asthma; Congestive heart failure; Coronary heart disease; COPD; Stroke; Gout; Cancer or Malignancy; BMI,kg/m²(Smooth); 25-hydroxyvitamin D3,nmol/L(Smooth); HDL cholesterol,mg/dL(Smooth); Triglyceride,mg/dL(Smooth); Glucose,mg/dl(Smooth); HbA1c,%(Smooth); Serum urate,mg/dL; eGFR,ml/min/1.73/m²(Smooth); AST,U/L(Smooth); ALT,U/L(Smooth); Thyroid problem .

Supplementary Table 4 Threshold effect analysis of serum vitamin B12 (pg/mL) and serum α-Klotho(pg/mL) levels using piece-wise linear regression. ( Non-vitamin B12 supplementation group)

| Models | Total | |  | Male | |  | Female | |
| --- | --- | --- | --- | --- | --- | --- | --- | --- |
|  | β (95%CI) | *P*-value |  | β (95%CI) | *P*-value |  | β (95%CI) | *P*-value |
| Model I |  |  |  |  |  |  |  |  |
| One line effect | 0.02 (-0.02, 0.05) | 0.0198 |  | 0.02 (-0.03, 0.07) | 0.4131 |  | 0.02 (-0.03, 0.07) | 0.4871 |
| Model II |  |  |  |  |  |  |  |  |
| Inflection point (K) | 899 |  |  | 631 |  |  | 984 |  |
| < K | 0.16 (0.09, 0.22) | <0.0001 |  | 0.26 (0.13, 0.38) | 0.0001 |  | 0.18 (0.10, 0.26) | <0.0001 |
| > K | -0.14 (-0.21, -0.07) | <0.0001 |  | -0.08 (-0.15, -0.01) | 0.0279 |  | -0.20 (-0.31, -0.10) | 0.0001 |
| Model fit value at K | 940.61 (915.30, 965.92) |  |  | 914.91 (888.08, 941.74) |  |  | 958.87 (919.65, 998.10) |  |
| LRT test | <0.001 |  |  | <0.001 |  |  | <0.001 |  |

Model Ⅰand Ⅱ represent one-dimensional linear regression and two-stage regression, respectively. Adjusted for: Age; Race; Alcohol use; Smoking status; Sleep disorder; Hypertension; Diabetes; Weak kidneys; Asthma; Congestive heart failure; Coronary heart disease; COPD; Thyroid problem; Liver condition; Stroke; Gout; Cancer or Malignancy; BMI; 25-hydroxyvitamin D3; HDL; Triglyceride; Glucose; HbA1c; Serum urate; eGFR; AST; ALT.


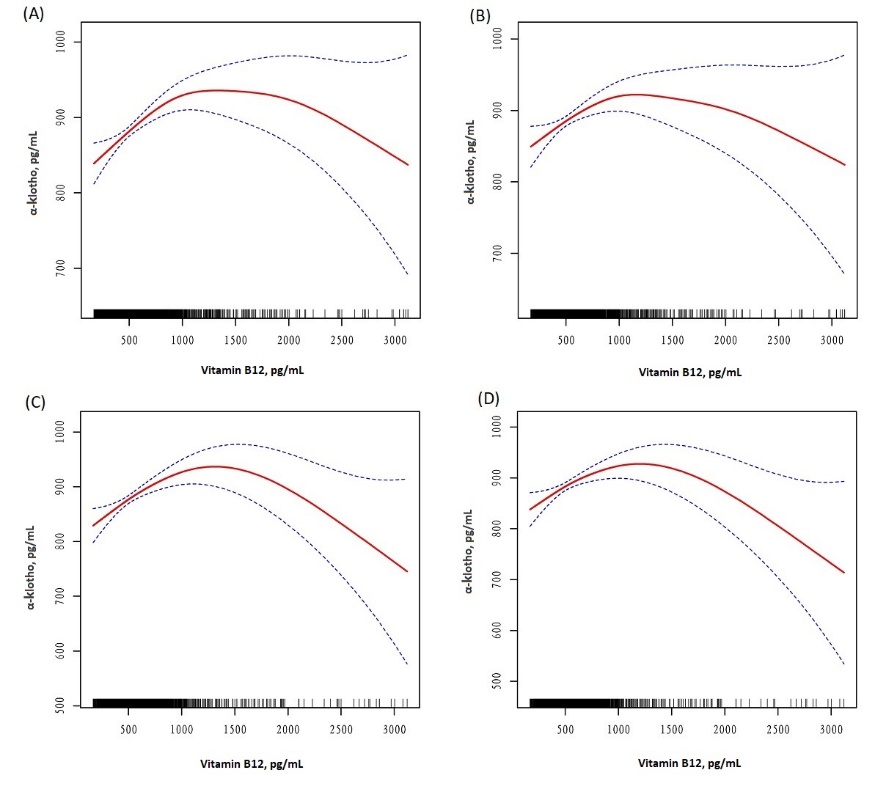


**Supplementary Figure** 1. (A and B, Age<60, years; C and D, Non-Hispanic White)

A threshold, a nonlinear association between the serum vitamin B12 (pg/mL) and serum α-klotho(pg/mL) levels was observed in the unadjusted model (A; C) (p<0.001) and adjusted model (B; D) (p<0.001), in a generalized additive model. The red and blue lines represent the smoothed curve fits between the variables and their 95% confidence intervals, respectively; In model B, all adjusted for Age; Race/Hispanic origin; Alcohol use; Smoking status; Sleep disorder; Hypertension; Diabetes; Weak kidneys; Asthma; Congestive heart failure; Coronary heart disease; COPD; Thyroid problem; Liver condition; Stroke; Gout; Cancer or Malignancy; BMI; 25-hydroxyvitamin D3; HDL; Triglyceride; Glucose; HbA1c; Serum urate; eGFR; AST; ALT. In model D, all of the above covariates were adjusted for except race.
